# An Internet-Based Real-Time Audiovisual Link for Dual MEG Recordings

**DOI:** 10.1371/journal.pone.0128485

**Published:** 2015-06-22

**Authors:** Andrey Zhdanov, Jussi Nurminen, Pamela Baess, Lotta Hirvenkari, Veikko Jousmäki, Jyrki P. Mäkelä, Anne Mandel, Lassi Meronen, Riitta Hari, Lauri Parkkonen

**Affiliations:** 1 Department of Neuroscience and Biomedical Engineering, Aalto University School of Science, Espoo, Finland; 2 MEG Core, Aalto Neuroimaging, Aalto University, Espoo, Finland; 3 BioMag Laboratory, HUS Medical Imaging Center, University of Helsinki and Helsinki University Hospital, Helsinki, Finland; CEA.DSV.I2BM.NeuroSpin, FRANCE

## Abstract

**Hyperscanning:**

Most neuroimaging studies of human social cognition have focused on brain activity of single subjects. More recently, “two-person neuroimaging” has been introduced, with simultaneous recordings of brain signals from two subjects involved in social interaction. These simultaneous “hyperscanning” recordings have already been carried out with a spectrum of neuroimaging modalities, such as functional magnetic resonance imaging (fMRI), electroencephalography (EEG), and functional near-infrared spectroscopy (fNIRS).

**Dual MEG Setup:**

We have recently developed a setup for simultaneous magnetoencephalographic (MEG) recordings of two subjects that communicate in real time over an audio link between two geographically separated MEG laboratories. Here we present an extended version of the setup, where we have added a video connection and replaced the telephone-landline-based link with an Internet connection. Our setup enabled transmission of video and audio streams between the sites with a one-way communication latency of about 130 ms. Our software that allows reproducing the setup is publicly available.

**Validation:**

We demonstrate that the audiovisual Internet-based link can mediate real-time interaction between two subjects who try to mirror each others’ hand movements that they can see via the video link. All the nine pairs were able to synchronize their behavior. In addition to the video, we captured the subjects’ movements with accelerometers attached to their index fingers; we determined from these signals that the average synchronization accuracy was 215 ms. In one subject pair we demonstrate inter-subject coherence patterns of the MEG signals that peak over the sensorimotor areas contralateral to the hand used in the task.

## Introduction

Social interaction constitutes an important part of human behavior, and its brain basis is under intensive study. However, neuroimaging studies of social cognition or social interaction have typically comprised just single participants at a time in carefully controlled but artificial environments, whereas experiments on complex and ecologically more valid social interactions between two or more subjects have been limited (for reviews, see [1–3]). To remediate this shortcoming, several research groups have started to employ hyperscanning—simultaneous neuroimaging of two or more interacting subjects, using functional magnetic resonance imaging (fMRI) [4], near-infrared spectroscopy (NIRS) [5], and electroencephalography (EEG) [6–12].

The fMRI community was the first to embrace the two-person neuroimaging approach starting with the seminal “hyperscanning” work by Montague et al. [4]. Simultaneous fMRI of two interacting subjects is an important methodological advance; nevertheless the inherent sluggishness of the haemodynamic response limits the usefulness of fMRI (and other haemodynamics-based modalities, such as NIRS) in studies of fast-paced social interactions, such as e.g. turn-takings during conversation.

EEG, on the other hand, provides millisecond-level temporal resolution necessary for probing the neuronal bases of fast social interaction. However, it only partially captures the available electromagnetic signatures of neuronal currents. Magnetoencephalography (MEG)—a method based on measuring the extracranial magnetic fields generated by neuronal currents—significantly complements EEG without compromising the temporal resolution (for a review, see [13]). MEG is less sensitive to inaccuracies in modeling the conductivity geometry between cortex and sensors. Moreover, in spatial localization accuracy, combined MEG–EEG measurements can outperform both, MEG and EEG alone [14].

However, unlike other neuroimaging modalities, MEG studies have mainly focused on single-subject recordings, with our previous study [15] and a more recent mother–child interaction study by Hirata et al. [16] as the only exceptions.

In our previous work we designed and validated an experimental setup that enables simultaneous MEG recording of two subjects connected with an accurate audio link based on a telephone landline [15], with lags of the order of 10 ms that would correspond the travel time for sound over a few meters and thus impossible for the subject to notice. In the current study, we extend our setup by adding a broadband Internet-based audio-video link, and report the results of a simple validation experiment.

## Methods

### Instrumentation

#### Overview

**Fig 1:**
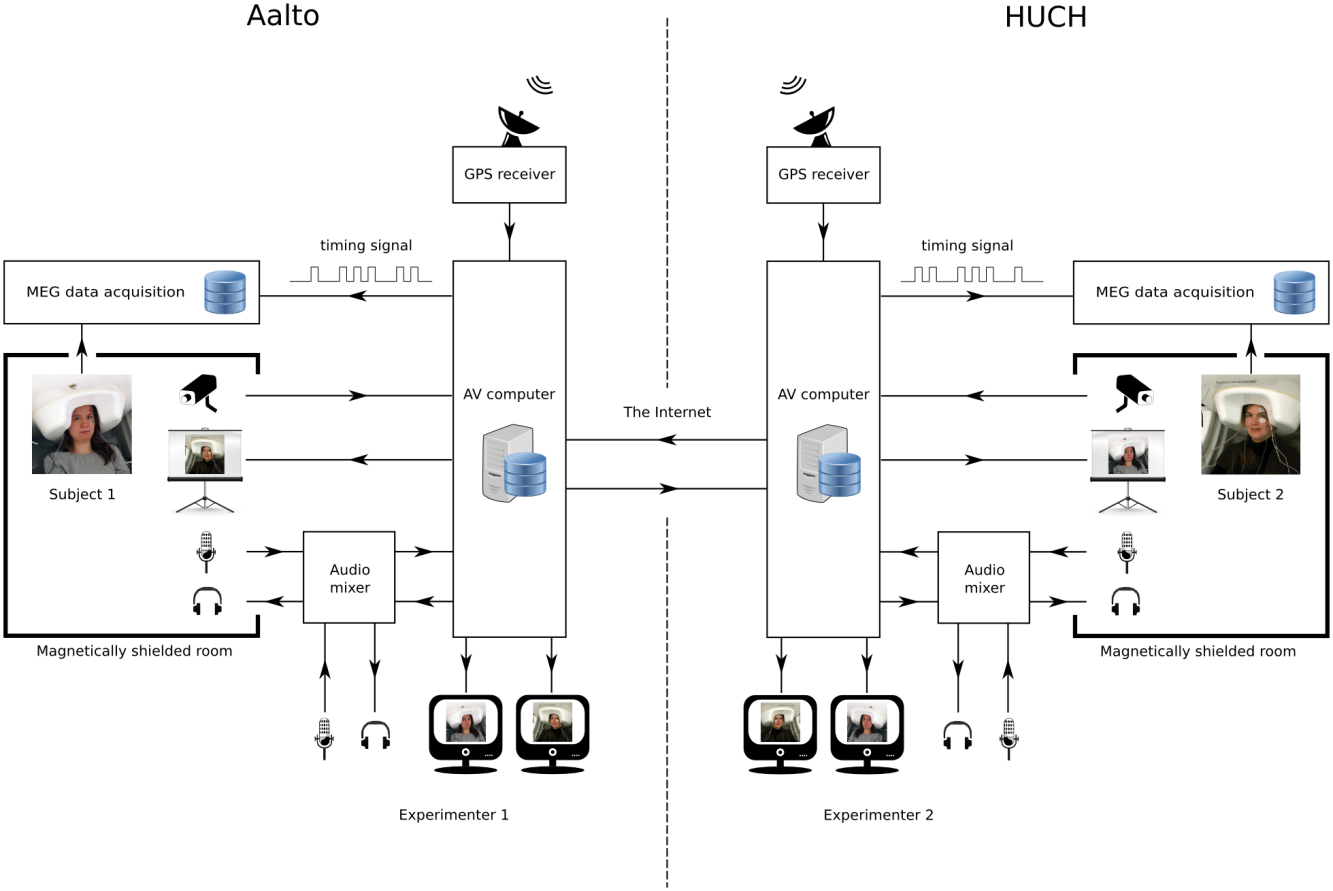
Schematic depiction of the experimental setup. The setup consists of two similar sets of hardware located at two different MEG sites linked over the Internet. The AV system (marked in red) allows the subjects to see and hear each other during the experiment. The experimenter at each site can monitor and instruct the subject at either site. The sites are synchronized by using GPS receivers that output timing signals.

Fig 1 shows the schematic diagram of our setup. We record MEG signals with two whole-scalp neuromagnetometers located at two different sites: one at the MEG Core, Aalto University School of Science, Espoo, Finland (hereafter referred to as Aalto), another at BioMag Laboratory, Helsinki University Central Hospital, Helsinki, Finland (hereafter referred to as HUCH). The distance between the sites is about 5 km. The subjects at the two sites interact with each other in real time via a custom-built audiovisual (AV) system. The AV system enables communication between the subjects as well as recording the audio and video streams at each site. For temporal co-registration, our setup brings all data streams (video, audio, and MEG) from both sites to a common timeline.

**Fig 2:**
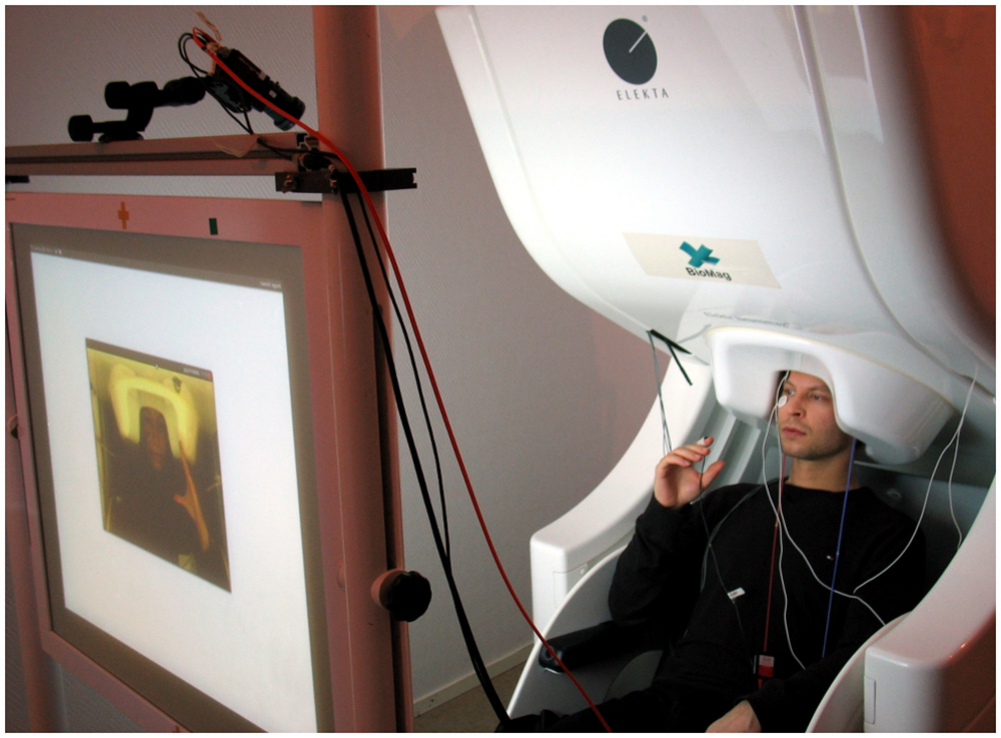
MEG2MEG setup at HUCH. The subject at HUCH performs a hand movement in synchrony with the subject at Aalto that can be seen on the backprojection screen. The subjects in the picture did not participate in the actual experiment.

Fig 2 shows that during the experiment the subject is seated inside the magnetically shielded room (MSR), with his head covered by the helmet-shaped neuromagnetometer. The subject from the other site is visible on the back-projection screen.

#### MEG recording

MEG signals are recorded with two 306-channel whole-scalp neuromagnetometers (Elekta Neuromag at Aalto and Neuromag Vectorview at HUCH; both are devices by Elekta Oy, Helsinki, Finland, with only minor differences in design). To reduce environmental noise, the devices are located inside high-quality MSR: a three-layer room by Imedco AG (Hägendorf, Switzerland) at Aalto, and a three-layer room by Euroshield/ETS Lindgren Oy (Eura, Finland) at HUCH. The devices have identical sensor arrays comprising 102 pairs of orthogonal planar gradiometers (204 in total) and 102 magnetometers.

#### AV system

The AV system allows the subjects and experimenters to interact in real time. The subjects can see and hear each other over the audiovisual link. At each site, the experimenter can monitor the video and audio from both sites and instruct the subjects. The system also allows documenting the interaction by recording the audio and video streams at each site. The system comprises two identical sets of hardware, one at each site. The hardware set includes:

1. A microphone and a set of MEG-compatible earphones for the subject located inside the MSR.
2. A microphone and a set of earphones for the experimenter located in the control room.
3. A full-matrix audio mixer that allows flexible routing of audio streams among the subject’s and experimenter’s microphones and earphones and the audio connections of the AV computer (described below).
4. A video camera and a standard video presentation setup (projector and back-projection screen) for capturing and presenting the video inside the MSR.
5. A computer that provides the videoconferencing, audio and video recording, and synchronization facilities (AV computer); this computer is connected to its counterpart at the other site via a standard Internet connection.
6. A GPS unit serving as an accurate time source.

#### Audio setup

We forfeited the optical microphone used for capturing the subject’s speech in our previous setup (Baess et al., 2012) in favor of a consumer-grade electret microphone that offers better sound fidelity. We present the audio to the subject through MEG-compatible insert earphones (Etymotic ER-2, Etymotic Research, Elk Grove Village, IL, USA). At each site the experimenters can interact with the subject through an additional set of headphones and a microphone located in the control room. As the AV computer’s audio interface we use E-MU 1616m digital sound card (E-MU Systems Inc., Scotts Valley, CA, USA) that allows low-latency (approximately 5 ms) audio capture and playback. The computer captures and plays back the audio at the sampling rate of 48 kHz. All the audio sources and destinations are connected to a full-matrix digital mixer (iDR-8; Allen & Heath, Cornwall, UK) that allows flexible routing of the audio streams according to the needs of the experiment.

#### Video camera

For video recording, we used Stingray F-033C machine-vision camera (Allied Vision Technologies GmbH, Stadtroda, Germany). The camera captures color video at the VGA resolution (640 by 480 pixels) at a rate of 15 to 30 frames per second (fps). The exact frame rate depends on the frame exposure time that is adjusted by the user. The camera is connected to the computer via an IEEE 1394 interface, also known as FireWire. The connection is physically implemented as an optical fiber, which reduces electromagnetic interference inside the MSR.

#### Video presentation

The AV computer software displays the video feed from the other site in a dedicated window. The computer is equipped with a video adapter with two outputs that are configured to produce identical outputs (“clone” mode). The outputs are connected to i) the monitor in the control room, and ii) the DLP projector (Panasonic PT-D7500E (Panasonic, Kadoma, Osaka, Japan) at HUCH; Panasonic PT-D7700E-K (Panasonic, Kadoma, Osaka, Japan) at MEG Core), outside the MSR projecting the video onto the back-projection screen (Elekta Oy, Helsinki, Finland) inside the MSR. The video adapter is configured to produce the signal at 60 fps, which is the native frame rate of the projector.

#### GPS time source

To accurately synchronize the AV computers, both sites use GPS (global positioning system) time sources. Each source consists of a custom-made GPS unit (at Aalto based on Fastrax uPatch100-S, u-blox, Thalwil, Switzerland, and at HUCH on Lassen iQ, Trimble Navigation Limited, Sunnyvale, CA, USA) with the antenna located within a clear view of the sky. The unit outputs a standard NMEA 0183 data stream and a pulse-per-second (PPS) signal. The PPS signal consists of square pulses; the rising edge of each pulse indicates the beginning of a new second of the GPS time. The PPS and NMEA signals are fed to the AV computer to synchronize its Network Time Protocol (NTP) server and real-time clock.

#### AV compute

The AV computer plays a central role in the setup, performing three main functions: (i) it provides a real-time audio–visual link between the two subjects, (ii) it records the video and audio streams, and (iii) it inserts timestamps into the recorded video, audio, and MEG data streams to allow for an accurate off-line temporal co-registration of all data. The software that provides this functionality has been developed in the framework of the Aalto MEG2MEG Project (supported by European Research Council Advanced Grant to R. Hari)—a collaborative open-source project aimed at providing MEG researchers with tools for conducting hyperscanning experiments [17]. It is distributed under the terms of GNU General Public License, version 3 [18] and is freely available from the project’s GitHub page. The software is written in C and C++ programming languages using Qt programming framework. It is based on the software for our experimental video-MEG system [19,20].

In our setup, the software is installed on a commodity office PC (Dell Optiplex 990, Dell, Round Rock, TX, USA) running the 64-bit version of Ubuntu 12.04 LTS Linux operating system.

#### Communication protocol

The AV computers send and receive audio and video streams using a simple custom-designed communication protocol implemented over User Datagram Protocol (UDP). We optimized the protocol for simplicity, short latency, and predictability of timing at the expense of optimal utilization of network bandwidth.

After receiving each video frame from the camera, the AV computer compresses it using the JPEG algorithm with the compression quality factor *q* = 60 and sends it to the other site in a single UDP packet, independently of other frames. Upon arrival of the packet, the AV computer at the other site decompresses the frame and displays it to the subject. The computer processes each frame immediately without any buffering or temporal reordering.

In a similar fashion, the sound card captures the audio signal, one frame (96 samples, corresponding to 2 ms of audio at our sampling rate of 48 kHz) at a time. The AV computer immediately sends each audio frame over the network uncompressed in a separate UDP packet. To reduce artifacts caused by a variable network delay, the receiving AV computer buffers the arriving frames. A buffer size of 15 audio frames turned out to be sufficient to provide good audio quality given our network connection.

During the experiment, the AV computer software does not check either audio or video streams for missing frames, out-of-order arrival, duplication or other problems that the UDP layer might introduce. However, the software assigns each frame a unique serial id number and timestamps it twice: once when being sent and once upon arrival at the other side. The AV computer records each frame’s id number and timestamps together with the frame data. Consequently, one can detect any problems in the network transmission off-line, during the analysis stage.

#### Synchronization mechanism

One of the tasks of the AV computers is to synchronize all data streams at both sites. To achieve this goal, we first synchronize the computers’ real-time clocks (RTCs) using the GPS time sources. Once the RTCs are accurately synchronized, they serve as master sources of timing information for all the data streams. The AV computers timestamp the video and audio streams by attaching the RTC time to every frame. To insert the timing information into the MEG data, each AV computer outputs serially-encoded timestamps via its parallel port into the MEG system’s trigger channel.

### System performance

Our setup allows videoconferencing between the two sites with one-way end-to-end latency of about 50 ms for audio and 130 ms for video over the current Aalto—HUCH network link. All the recorded video, audio, and MEG data streams are aligned with an accuracy of 1 ms. For more details on quantifying the system performance, see S1 Appendix.

### Validation experiment

To validate our setup we performed a simple experiment in which the subjects used the video link to synchronize their hand movements.

#### Experimental setup

Nine pairs of subjects (12 males, 6 females; mean ± SD age 27.1 ± 5.6 years, range 21–43 years) with normal or corrected-to-normal vision participated in the experiment. The subjects gave their written informed consent after the course of the study had been explained to them. The study had prior approval by the Ethics Committee of the Hospital District of Helsinki and Uusimaa and all participants gave their written informed consent before the experiment. The individuals appearing in Figs 1 and 2 have given written informed consent for the publication.

**Fig 3:**
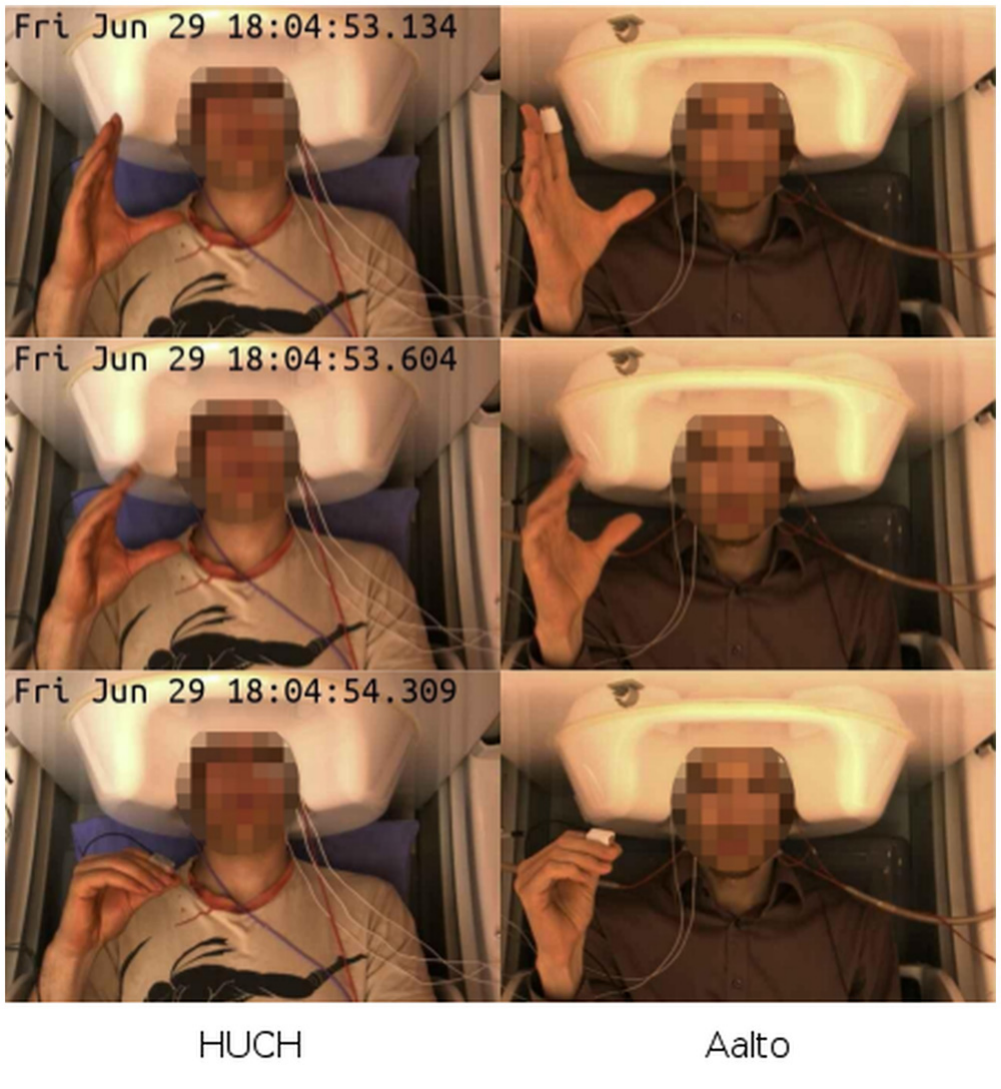
Three video frames showing two subjects mirroring each other’s hand movements. Subjects were synchronizing their movements by observing each other via the video link.

We asked the subjects of each pair to perform self-paced repetitive movements of the right-hand fingers against the thumb for 5 min so that the movements between the participants were as synchronized as possible; importantly, there was no predefined leader for the task. To achieve synchronization, the subjects observed each other over the video link (see Fig 3; S1 Video).

We recorded MEG signals from both subjects with sampling frequency of 1 kHz. Additionally, we monitored the hand movements with 3-axis accelerometers (ADXL335 iMEMS Accelerometer, Analog Devices, Inc., Norwood, MA, USA) attached to the subjects’ index fingers. For technical reasons at the Aalto site, we recorded the accelerometer signals with MEG analog inputs in 3 subject pairs and with EEG input of the MEG device for 6 subject pairs. At HUCH, we recorded the accelerometer signals with the MEG analog inputs for all subjects. We also recorded the video of the subjects at both sites.

The anonymized accelerometer data are available as supplementary material. Due to the national legislation regulating research on human subjects, we are not able to distribute the original MEG data.

#### Behavioral data analysis

For each pair of subjects, we synchronized the video streams recorded at the two sites and merged these into a single video file that displayed the two subjects side-by-side (see Fig 3; S1 Video). We reviewed the resulting video file to verify that the subjects performed the task correctly. To discard any transient failures in performance at the beginning or end of the task, we restricted our analysis to a single continuous 4-min block of data. For each subject pair, we manually selected the block location so that it did not contain any transient task failures. To remove power-line interference, we notch-filtered the accelerometer signals at 50 Hz using a second-order Infinite Impulse Response (IIR) filter with a bandwidth of 1 Hz at –3 dB. To avoid any phase distortion, the filter was applied to the signals twice, once in forward and once in reverse direction.

**Fig 4:**
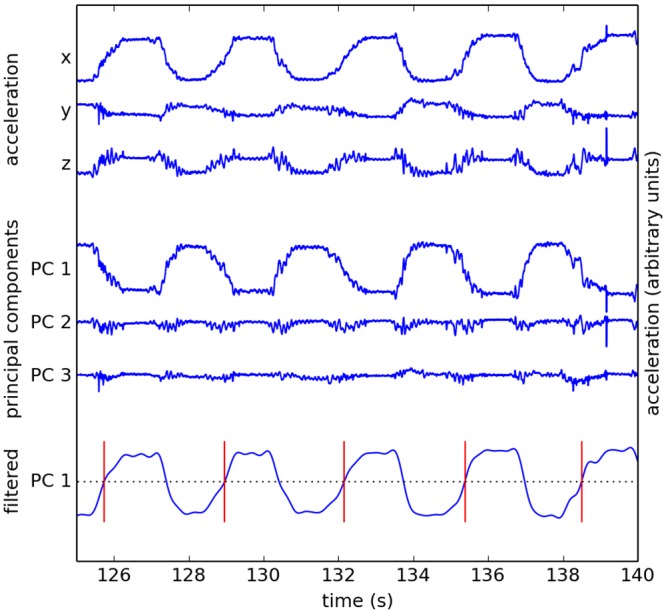
Detecting events from accelerometer signals. Acc x, Acc y, and Acc z are the signals from the orthogonal x, y, and z accelerometers. PC 1, PC 2, and PC 3 are the principal components in the order of decreasing variance. LP is the low-pass-filtered (at 3 Hz) version of PC 1. Vertical red markers denote the detected events i.e. the rising zero-crossings of LP.

Fig 4 illustrates the three components of the accelerometer signals and the stages of their analysis. The finger movements performed by the subjects had essentially a single degree of freedom. Thus, we wanted to reduce the 3-dimensional accelerometer data to a single summary signal that captured the relevant aspect of the movement. We selected Principal Components Analysis (PCA) as the reduction method because it is simple and easy to interpret, and because it provides a uniform representation of the movement that is invariant, e.g., to accelerometer orientation. Moreover, visual inspection of the principal components (PCs) of all the participants indicated that the first principal component (PC1) reliably captured most of the movement (see, for an example, the middle rows of Fig 4).

We performed PCA for each subject and selected PC1 for further analysis. Since principal components are defined up to an arbitrary multiplicative factor of ±1, we multiplied the largest principal components from all 9 subject-pairs by 1 or –1 as necessary to ensure that the resulting waveforms exhibited the same polarity for all subjects. We then low-pass (LP) filtered PC1 at 3 Hz using Type II Chebyshev IIR filter of order 6 with stopband attenuation of 30 dB from the peak passband value (the bottom row in Fig 4). We chose the Chebyshev Type II filter to avoid distortion in the passband. Similarly to the notch filter, the LP filter was applied twice, once in forward and once in reverse directions. We marked each time point when the LP signal crossed zero from negative to positive values as an event (vertical red lines). For each subject pair, we detected an identical number of events at the two sites (in one case we had to adjust the location of the 4-min block by less than 1 s to make sure that the matching events from the both sites are properly included).

#### MEG data analysis

In addition to the behavioral data analysis, we conducted connectivity analysis of the MEG signals between the subjects of one pair. We used coherence as the connectivity measure as it robustly detects relations between MEG signals and hand acceleration [21]. Our subjects were able to accurately synchronize their hand movements, as measured by accelerometers (see the Results section), and we expected this synchronization to mediate sensor-level coherence between the two subjects’ MEG signals in a frequency band that includes the movement frequency.

We first reduced the external interference by applying signal-space separation (SSS) method [22] to the MEG signals (MaxFilter software version 2.2.10; Elekta Oy, Helsinki, Finland).

As a proof-of-concept, we only analyzed the data of pair #3 that had the largest number of repetition cycles and a low variability of the cycle length. The analysis involved the 204 planar first-order gradiometers that in our neuromagnetometers are arranged in 102 modules of 2 gradiometers each. The gradiometers in one module correspond to the same location on the helmet surface and measure the orthogonal components of the magnetic field’s gradient.

We estimated magnitude-squared coherence between the gradiometer signals of the two subjects in a pairwise fashion, resulting in a 204-by-204 coherence matrix that was computed using the same 4-min block of data that were used for the behavioral analysis. The rows correspond to the gradiometers at HUCH and the columns to the gradiometers at Aalto. The entry (*i*,*j*) of the matrix contains the estimate of the magnitude-squared coherence between the signals from the *i*-th gradiometer at HUCH and *j*-th gradiometer at Aalto. We estimated the average coherence in the 0.5–2 Hz frequency window with Welch’s periodogram method using window length of 8096 samples (approximately 8 s) which provided about 0.12-Hz frequency resolution.

We then averaged the coherence matrix of the subject pair separately along the rows and columns. The resulting 204-dimentional vectors describe the distribution of the average coherence over the HUCH and Aalto gradiometers, respectively. To visualize the results, we further averaged the coherence over the two gradiometers in each module. That way we obtained two 102-dimensional vectors that characterize the distribution of average coherence over the helmet surface for both participants. We then plotted these vectors on flattened helmet surface. Data analysis was performed in MATLAB R2014b and Python version 2.7.6 software. We used MNE software [23] (version 2.7.4) and MNE-Python [24] (version 0.9) for importing the MEG data, and we visualized the results using MNE-Python and Matplotlotlib library [25] (version 1.3.1).

The source code for the data analysis is available in S2 Appendix. Anonymized accelerometer data is available in S3 Appendix.

## Results

Reviewing the videos revealed that every subject pair was able to perform the task for at least 4 min. None of the subject pairs reported any difficulties during the experiment.

### Behavioral data analysis

**Fig 5:**
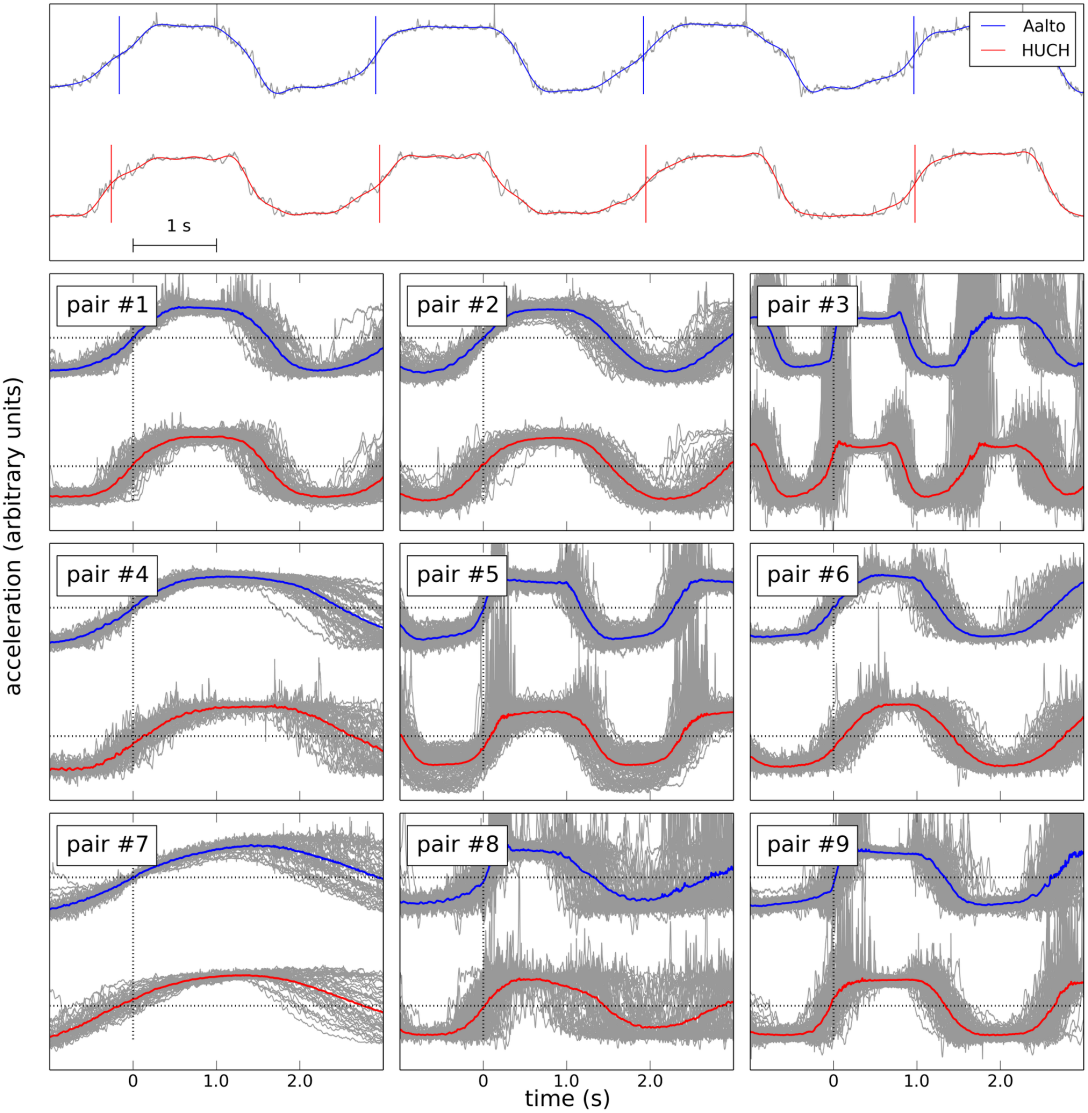
Accelerometer signals. The upper box shows the first principal components from the two sites. The plot depicts the original, unfiltered signals (grey) and the same signals low-pass filtered at 3 Hz (red—HUCH, blue—Aalto). The detected events are marked by vertical lines. The lower nine boxes depict segmented first PC for the nine subject pairs. Grey lines denote individual movement cycles time-locked to the events detected at Aalto. The upper traces are from Aalto, the lower from HUCH. The thick blue and red curves show the averages for Aalto and HUCH, respectively.

The upper box of Fig 5 provides a characteristic example of PC1 from the two subjects and the corresponding events (red and blue vertical markers). The rising zero-crossings were reliably detected and matched between the sites. We quantified the behavioral synchronization accuracy in two ways: (1) Segmenting and averaging the full-bandwidth accelerometer signals at one site time-locked to the events detected on the other (time window from –1 to 3 s around the trigger; no baseline correction), and (2) computing time differences between matching events between the two sites.

We were able to reliably match all the events for all subject pairs (the time difference between the matched events was always smaller than one third of the movement cycle duration—the difference between two consecutive events at the same site).

**Fig 6:**
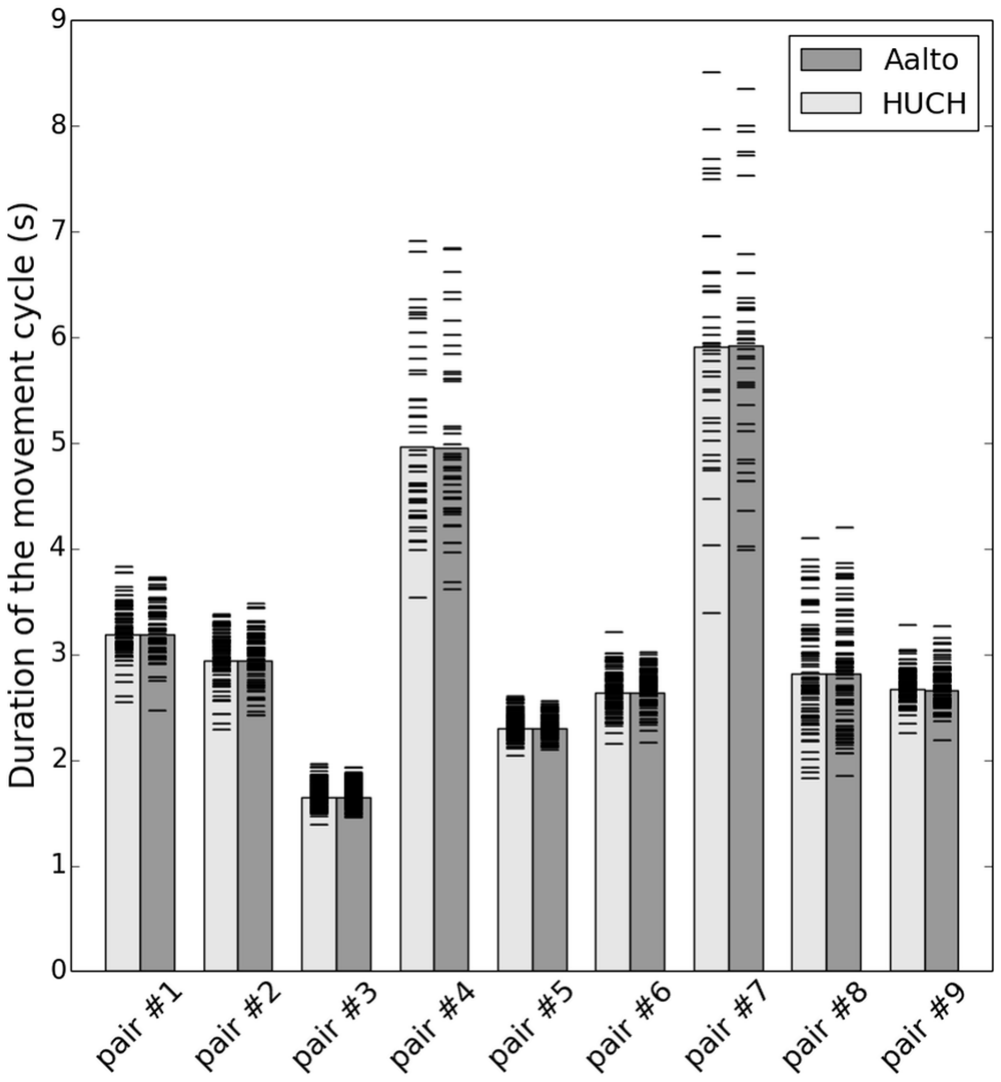
Pairwise comparison of the lags between HUCH and Aalto. The upper boxplot shows the distributions of the signed lags, the lower the distribution of the absolute values of the lags. The red line indicates the median delay for subject each pair, the box the location of the 25-th and the 75-th percentiles, and the whiskers the minimum and maximum delay values.

Over the 4-min period the subjects performed between 39 (pair #7) and 144 (pair #3) movement cycles, the average number of cycles per pair was 83.4. The average duration of a movement cycle for a given pair of subjects varied from 1.7 s for the fastest pair to 5.9 s for the slowest, corresponding to movement rates of 0.17–0.60 Hz (see Fig 6). The longest cycle lasted 8.5 s (pair #7, HUCH subject) and the shortest 1.4 s (pair #3, HUCH subject).

All subject pairs maintained a relatively constant movement rate throughout the experiment, which is reflected as the relatively narrow dispersion of individual cycle durations around the average (see Fig 6); no single cycle duration deviated from the average by more than 50%.

The lower 9 boxes of Fig 5 describe the movement synchrony between the subjects in all the nine pairs. The superimposed segmented signals demonstrate a similar behavioral pattern at both sites, both at the level of single segments and averages.

**Fig 7:**
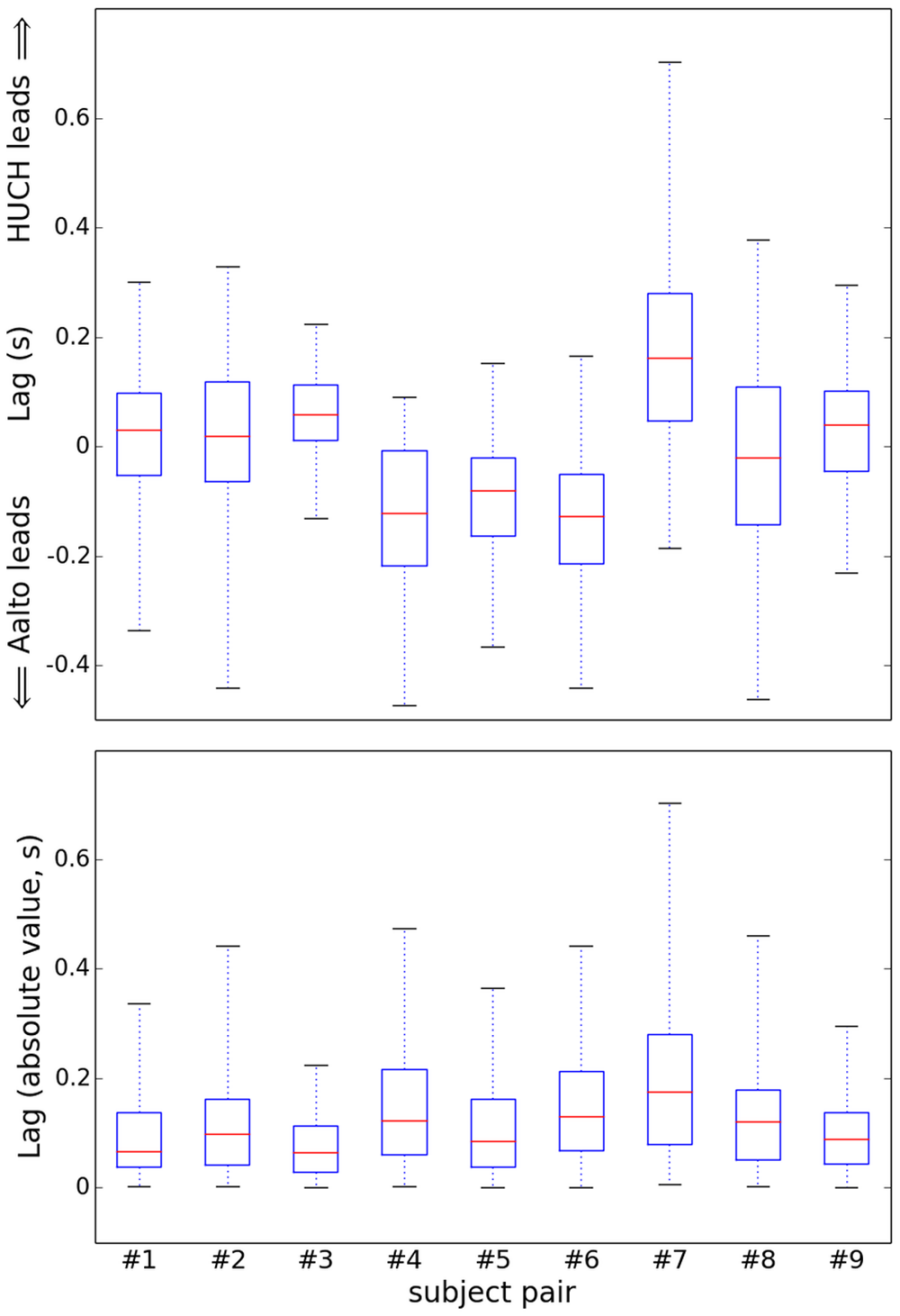
Rate of movement (duration of one movement cycle) for all nine subject pairs. For each subject pair, the bar denotes the average cycle duration at Aalto (dark) and at HUCH (light). The small horizontal lines mark the durations of the individual cycles.

Fig 7 presents the lags between the subjects computed by matching each event at one site to the temporally closest event at the other site; the upper box—the signed lag and lower—the absolute value. All subject pairs were able to achieve synchronization accuracy of 215 ms or better (in terms of average absolute lag) with the best pair (#3) attaining the accuracy of 77 ms.

### MEG data analysis

**Fig 8:**
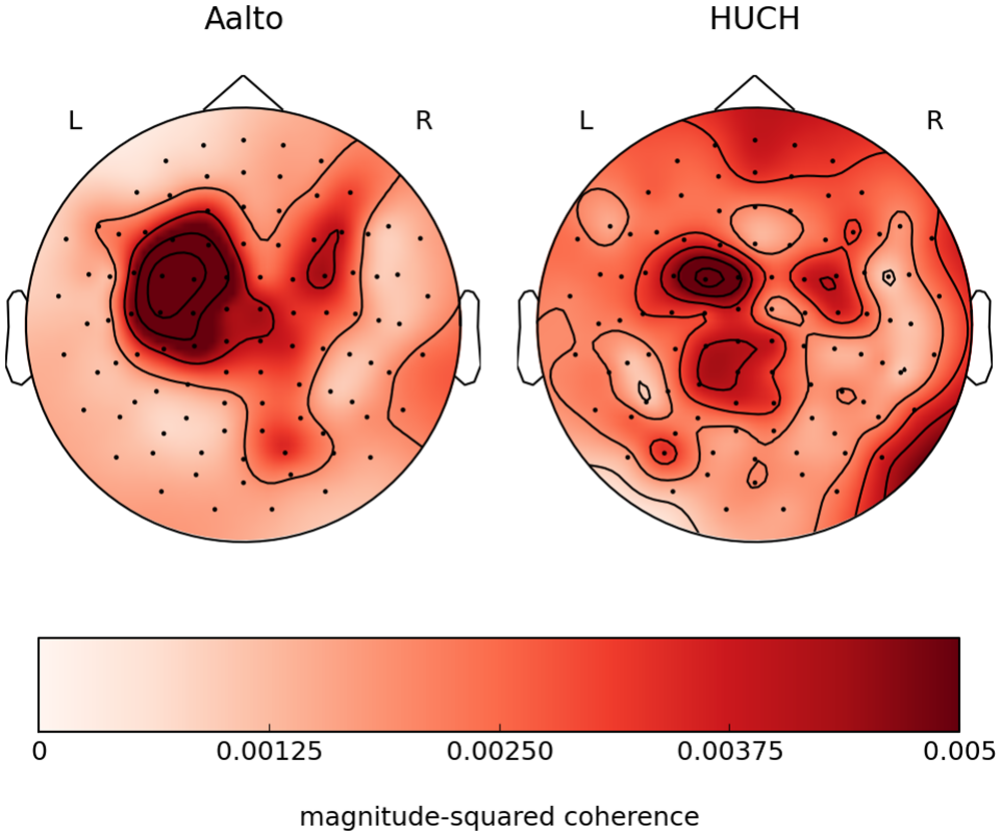
Average inter-subject coherence for subject pair #3. The flattened sensor-helmet maps show the average coherence in the 0.5–2-Hz band for one pair of subjects.

Fig 8 depicts the average coherence distribution over the MEG helmet for the subjects of pair #3. The map exhibits a clear maximum over the contralateral sensorimotor area. Relatively low coherence values can be explained by the fact that for each location the map depicts an average coherence value to all the channels from the other site, most of which are unrelated to the given location. The maximum value of magnitude-squared coherence for a single pair was approximately 0.06.

## Discussion

One of the main obstacles to MEG hyperscanning is the price of MEG systems, including the shielded room, so that no laboratory at present has two identical MEG devices under the same premises. Unlike, for example, EEG, where signals can be easily recorded from multiple participants in the same room, MEG hyperscanning (with one exception so far [16]) needs to involve two MEG devices located at two geographically separate sites. Such a setup requires an accurate audiovisual link enabling the two subjects to interact and an accurate procedure for synchronizing the two MEG recordings.

We started building an MEG-to-MEG two-person recording setup by first implementing an audio-only link that used telephone landlines and that thus allowed us to achieve a low-latency (12.7 ms) connection with negligible transmission time [15]. However, the setup naturally limited the possible experiments to those relying only on auditory interaction.

In our current audiovisual setup, we shifted to the Internet as the underlying communication channel. This transition comes at a price of longer transmission delays and increased jitter. The jitter can be to a considerable degree mitigated by buffering at the receiving site, thus trading additional delay for reduced jitter. The key question is therefore whether an Internet link can provide a level of performance necessary to take the full advantage of MEG’s high temporal resolution in two-person recordings. Our validation experiments provide some insights into this question.

The measured one-way delays, 50 ms for the audio and 130 ms for the video streams, are in line with the requirements of less than 100-ms (audio) and 500-ms (video) delays mediating smooth social interaction [26,27]. The 80-ms misalignment between the audio and video streams falls within the perceptual integration window of natural speech [28,29]. Consequently, all our subject pairs were able to effortlessly maintain a free conversation over the link and to perform the finger-movement synchronization task without perceiving any timing problems introduced by the setup.

The results of the behavioral validation experiment further indicate that our setup can be used for investigating the mechanisms of motor synchronization between subjects. All the subject pairs succeeded in the task. It is notable that some subject pairs (e.g. pair #3) managed to synchronize their behavior so accurately that the lag between the subjects’ movements was considerably shorter than the AV delay. Such accuracy obviously reflects mutual adaptation, based on predictive models of the partner’s behavior, rather than simple leader/follower synchronization.

Our MEG data analysis produced subject-to-subject coherence maps that showed, in both subjects, clear maxima over the sensorimotor area contralateral to the moving hand. It was previously demonstrated that for a single participant, the distribution of coherence between the MEG signals and the hand acceleration peaks over the contralateral sensorimotor cortex [21]. If two participants synchronize their hand accelerations accurately enough, one expects to find similar distribution of coherence between each participant’s MEG sensors and the peer’s hand acceleration, and, by the transitivity of coherence, between the two participants’ MEG sensors. Thus our findings are in line with results previously reported for single subjects, meaning that our setup can not only mediate sub-second behavioral synchronization between subjects, but also synchronization of the subjects’ brain signals that are associated with such joint behavior.

## Conclusions

We have demonstrated the feasibility of building a multi-site MEG setup for probing the brain mechanisms of human interaction mediated via visual and auditory channels. Our setup relies on widely available off-the-shelf components and a standard Internet connection. It achieves performance sufficient for investigating interactions occurring at timescales from tens to hundreds of milliseconds, which allows the researcher to exploit the high temporal resolution of MEG.

## Supporting Information

S1 Appendix Quantifying the dual MEG system performance. (DOC)

S2 Appendix Source code for the data analysis. (ZIP)

S3 Appendix Accelerometer data. (ZIP)

S1 Video Subjects synchronizing their hand movements during the validation experiment. (AVI)
