# Supplementary material for: An Internet-Based Real-Time Audiovisual Link for Dual MEG Recordings
Source: PLoS One. 2015 Jun 22;10(6):e0128485. doi: 10.1371/journal.pone.0128485 (PMC4476621; doi:10.1371/journal.pone.0128485)

# Appendix S1: Methods for quantifying synchronization accuracy and latency

**Synchronization of MEG signals.** To measure the accuracy of the synchronization of MEG signals, we used the Pulse-Per-Second (PPS) output of the GPS receivers installed at both sites; the rising edges of the PPS pulses are synchronized with sub-microsecond accuracy by the GPS system and therefore provide accurate enough reference for our measurement. At both sites, we injected the PPS signal into an analog input channel of the MEG acquisition system. After the recording, based on the timestamps inserted into the trigger channel, we determined the time at the rising edge of each PPS pulse and compared these times between the two sites. The accuracy of synchronization was about 1 ms.

**Figure S1:** Setup for the end-to-end latency measurement.


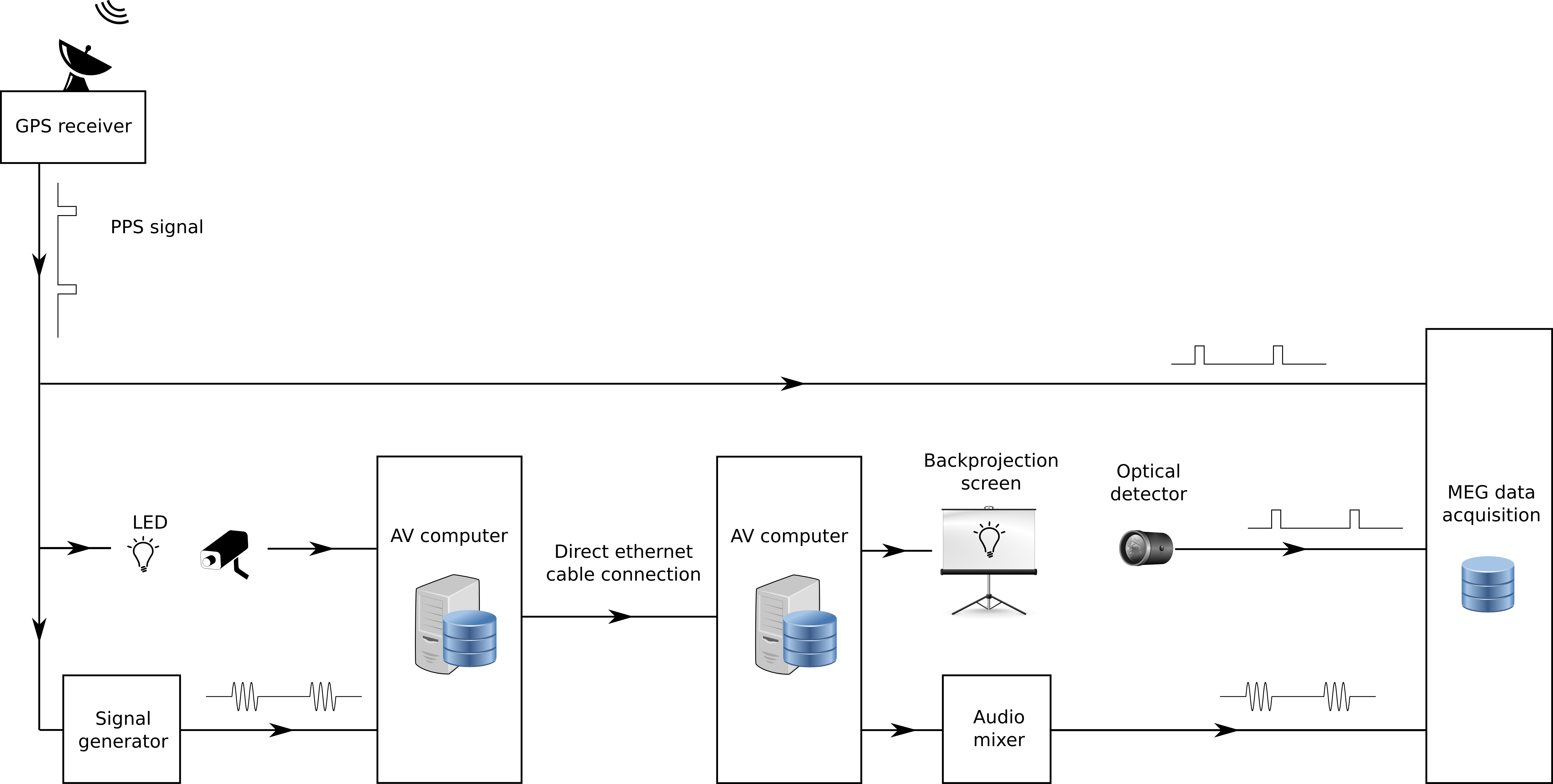


**End-to-end latency measurement.** To characterize the AV system performance, we performed an end-to-end measurement of the video and audio signal propagation times. Figure S1 depicts the experimental setup. For this measurement, we used a more recent version of the AV software than the one used in the behavioral validation experiment. However, the differences between the two versions do not involve any components that affect the timing of video and audio transmission.

Delays can arise from two sources: (1) the inherent delay caused by the peripheral devices (sound cards, cameras, projectors, etc.) and the processing in the AV computers, and (2) the network delay caused by the transmission time of the UDP packets sent between the sites over the Internet. The former is independent of the network characteristics while the latter varies depending on the network connection and load. To isolate the inherent delay, we measured the audio and video propagation times between two AV computers located at the same site and connected directly to each other with a network cable. This arrangement ensured sub-millisecond UDP packet propagation delays.

The rising edge of the PPS signal triggered a LED flash and a short beep (140 Hz, 200 ms, abrupt onset and offset). The sending AV computer captured the flash through the camera, and the sound pip was fed directly (without a mixer) into the AV computer’s audio input. The computer transmitted the audio and video signals to the receiving computer. We projected the video onto the screen inside the magnetically shielded room and detected the flash with a custom-built optical detector placed on the screen and recorded with an analog input channel of the MEG device. We routed the audio signal from the receiving computer through the mixer to another analog input channel of the MEG system. The PPS signal was recorded in the trigger channel.

Except for the network part, our latency measurement mimicked the video delays of the real experiments. In the audio delay measurement, we omitted the contribution of the tubes used for delivering the sound to subject's ears and the delay of the iDR-8 mixer at the sending site, which together correspond to an additional delay of about 5 ms.

Over a period of approximately 40 minutes we measured the delays for 2424 LED flashes and 2424 audio beeps to characterize video and audio latencies. The results are summarized in Figure S2. The end-to-end latency for audio was about 50±2 ms and for the video about 130±12 ms (mean±standard deviation).

**Figure S2:** Propagation delays for the audio and video streams. For the video, the delay was computed for each frame, for the audio – for each 2 ms long chunk. The histogram summarizes the results.


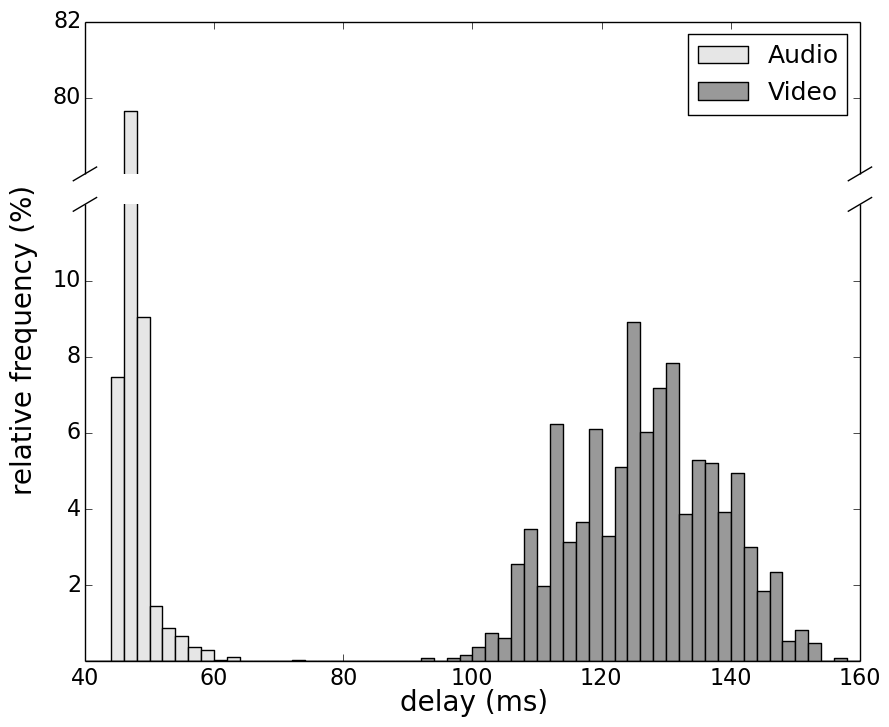

Supplement: S1 Appendix — (DOC) [file pone.0128485.s001.doc]
